# OA12 Dactylitis in PsA: aetiology, clinical significance, & treatment implications

**DOI:** 10.1093/rap/rkac066.012

**Published:** 2022-09-28

**Authors:** Andrew Melville

**Affiliations:** University of Glasgow, Glasgow, United Kingdom

## Abstract

**Introduction/Background:**

Dactylitis is a hallmark feature of PsA (PsA) and related spondyloarthritides and may affect up to half of PsA patients during the course of their disease(1). Presence of dactylitis may imply a more aggressive disease phenotype; dactylitis at presentation is associated with higher swollen and tender joint counts, higher systemic inflammatory response, presence of ultrasound-detected inflammation and erosions(2), as well as radiographic joint progression(3). Trial data suggest treatments used in PsA may not be equally effective against dactylitis(4).

**Description/Method:**

A 55-year-old man was referred to rheumatology with several months of pain in the right index finger. He was unable to hold a pen, use a computer, or play golf. He had chronic plaque psoriasis since late 20s. On examination, right index finger was mildly swollen, suggestive of dactylitis, and flexion was restricted. No other joints or digits were affected. Inflammatory markers and x-rays of hands and feet were normal. A diagnosis of PsA was made. He had been reviewed by dermatology a few months earlier and started on apremilast; his finger seemed to be improving, so apremilast was continued and etodolac added PRN.

Over the next 2 years he reported short-lived episodes of finger pain but had no objective abnormalities when assessed in clinic. He then developed more persistent left hand pain and stiffness, was felt to have wrist and MCP synovitis, and started on sulfasalazine 1.5g daily, with symptomatic improvement.

Two years later he reported pain in the right hand, with inability to make a fist or play golf. Clinically he had synovitis at the right 4^th^ PIPJ, and soft tissue swelling affecting the 2^nd^ and 3^rd^ fingers. He was given an IM glucocorticoid and sulfasalazine dose was increased to 3g daily. After 3 months he had ongoing difficulty bending the right 4^th^ finger, and mild proximal swelling, and was referred for ultrasound. This showed PIP synovial hypertrophy, an inflamed extensor tendon, marked flexor tenosynovitis, and soft tissue swelling, consistent with dactylitis. He underwent guided injection to the flexor tendon sheath. Four weeks later he reported complete resolution of pain, and 90% improvement in swelling and function. Very recent x-rays of hands and feet showed no visible erosions in the hands, but a large juxta-articular erosion in the right middle toe.

**Discussion/Results:**

This is a case of PsA characterised by isolated finger dactylitis at presentation, and dactylitis as a prominent feature of flare over time.

In general, dactylitis is more common in feet than hands, implicating mechanical stress as a key aetiopathogenetic driver. This patient was a keen golfer, which may explain predominant involvement of his right (dominant) hand 2^nd^, 3^rd^ and 4^th^ fingers.

While dactylitis is a key disease domain in PsA and other spondyloarthritides, it is not specific to these conditions, and other differentials should be considered depending on specific context, e.g. soft tissue infection or gout. While the diagnosis of PsA seems well-established here, a recent foot x-ray showed a middle toe punched out juxta-articular inter-phalangeal erosion, more typical of gout.

As well as an indicator of arthritis severity and a poor prognostic factor for radiographic progression, the number of dactylitic digits has been shown to be associated with major cardiovascular events, independent of traditional risk factors(5). Presence of dactylitis should perhaps prompt particularly careful assessment of cardiovascular risk.

Recent international (GRAPPA) guidelines give strong recommendations for all targeted therapies commonly used in PsA, including anti-TNF, anti-IL17, anti-IL12/23, anti-IL23, JAKi, and apremilast. NSAIDs, local steroid injections and methotrexate are conditionally recommended “for”, while other csDMARDs are conditionally recommended “against”(4). Whether sulfasalazine is truly less effective in this specific disease domain, or this simply represents a lack of supporting evidence, is debatable. In this case, the combination of apremilast and sulfasalazine was reasonably successful, but further flares might warrant a change in therapy, taking account of the dactylitis history.

In cases of uncertainty, ultrasound can be useful for confirmation of dactylitis(6), and/or differentiating between acute (“hot”) dactylitis and chronic (“cold”) dactylitis. The involvement of multiple structures and soft tissues can be visualised and appreciated.

**Key learning points/Conclusion:**

Dactylitis is a hallmark feature of PsA and may be the sole musculoskeletal manifestation. Mechanical stress appears to be an important factor in aetiopathogenesis. Differentials others than spondyloarthritis should be considered. Presence of dactylitis tends to imply a more aggressive PsA phenotype and may have clinical relevance beyond the joints, including increased cardiovascular risk. Assessment for dactylitis should be performed when evaluating disease activity across psoriatic disease domains, and presence of dactylitis incorporated into decisions about treatment.
